# Successful Minimally Invasive Mitral Valve Repair and Maze Procedure in a Patient With Myotonic Dystrophy

**DOI:** 10.7759/cureus.109598

**Published:** 2026-05-25

**Authors:** Takeshi Kakiuchi, Rie Iwasaki, Takahiro Yamamoto, Daijiro Hori

**Affiliations:** 1 Cardiovascular Surgery, Ageo Central General Hospital, Saitama, JPN

**Keywords:** anesthesia, minimally invasive cardiac surgery, myotonic dystrophy type 1, perioperative care, respiratory function

## Abstract

Myotonic dystrophy type 1 (DM1) is an autosomal dominant multisystemic disorder caused by unstable nucleotide repeat duplications. Management of general anesthesia is challenging due to the high risk of myotonia and postoperative respiratory complications. While few cases of cardiac surgery in patients with DM1 have been reported, there is no reported case of utilizing minimally invasive cardiac surgery (MICS), which avoids sternotomy for preservation of respiratory function in these patients. We report a successful case of MICS mitral valve repair (MVr) and a maze procedure in a patient with DM1. To minimize postoperative respiratory depression, a paravertebral block was also employed, which successfully reduced opioid dosage. In patients with myotonic dystrophy, an MICS approach combined with cautious perioperative pharmacological management and peripheral nerve blocks may effectively mitigate the risk of postoperative respiratory complications.

## Introduction

Myotonic dystrophy types 1 (DM1) and 2 (DM2) are autosomal dominant genetic disorders caused by the unstable expansion of nucleotide duplications. Clinical manifestations are multisystemic and include distal to proximal muscle weakness, myotonia, respiratory failure, early-onset cataracts, cardiac arrhythmias, and cognitive impairment [1]. Cardiac involvement is a major cause of morbidity and mortality in these patients, and conduction abnormality, as well as valvular disease, may require intervention. It is the most common and severe form of myotonic syndrome with an estimated incidence of 9.27 cases (95% confidence interval: 4.73-15.21) per 100,000 [2].

Management of patients with myotonic dystrophy under general anesthesia requires careful attention to postoperative respiratory complications and the occurrence of myotonia [3]. Respiratory muscle weakness and impaired cough clearance may predispose these patients to prolonged ventilation and postoperative pulmonary complications after median sternotomy, making perioperative management complex in these patients. Although isolated reports of conventional cardiac surgery in DM1 exist, evidence regarding minimally invasive valve surgery and perioperative respiratory preservation strategies remains extremely limited. To the best of our knowledge, there has been no reported case of myotonic dystrophy patients undergoing minimally invasive cardiac surgery (MICS). No records are found in the PubMed search using the words "MICS" and "myotonic dystrophy."

Here, we report a successful case of MICS-MVr and a Maze procedure in a patient with myotonic dystrophy and severe mitral regurgitation.

## Case presentation

A 50-year-old male (height: 176 cm, weight: 70 kg, body mass index: 24.2 kg/m²) presented to our department with a chief complaint of palpitations and dyspnea. He first noted myotonia and muscle weakness in both upper extremities in his mid-30s. At age 45, genetic testing revealed 1,150-1,600 cytosine-thymine-guanine (CTG) duplications, confirming the diagnosis of DM1. At that time, his grip strength was reduced to 5 kg in both hands, and his Lawton instrumental activities of daily living (IADL) scale was 3 points; however, he maintained an independent lifestyle with the assistance of home care services.

**Figure 1:**
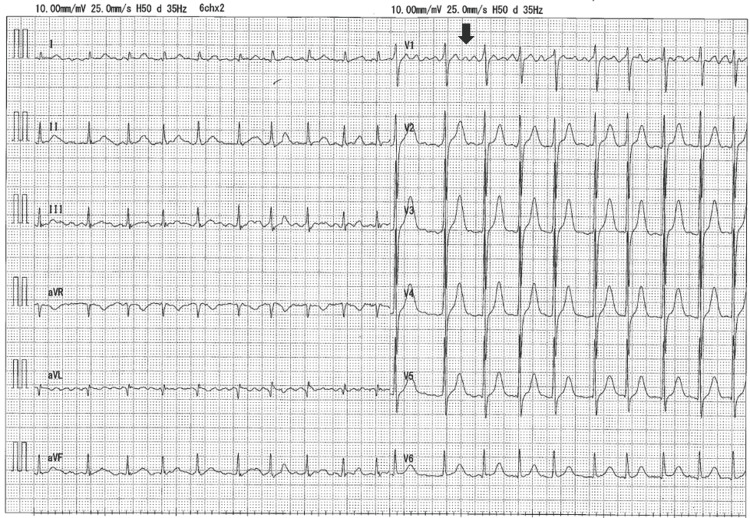
Electrocardiography Preoperative electrocardiography showing atrial fibrillation. The arrow shows the f wave.

Although severe mitral regurgitation (MR) was already identified at age 45, he remained asymptomatic and was managed by medication. At age 50, the patient developed new-onset atrial fibrillation (AF), which led to his referral to our hospital. Electrocardiography demonstrated AF with a heart rate of 116 beats per minute (Figure 1).

**Figure 2:**
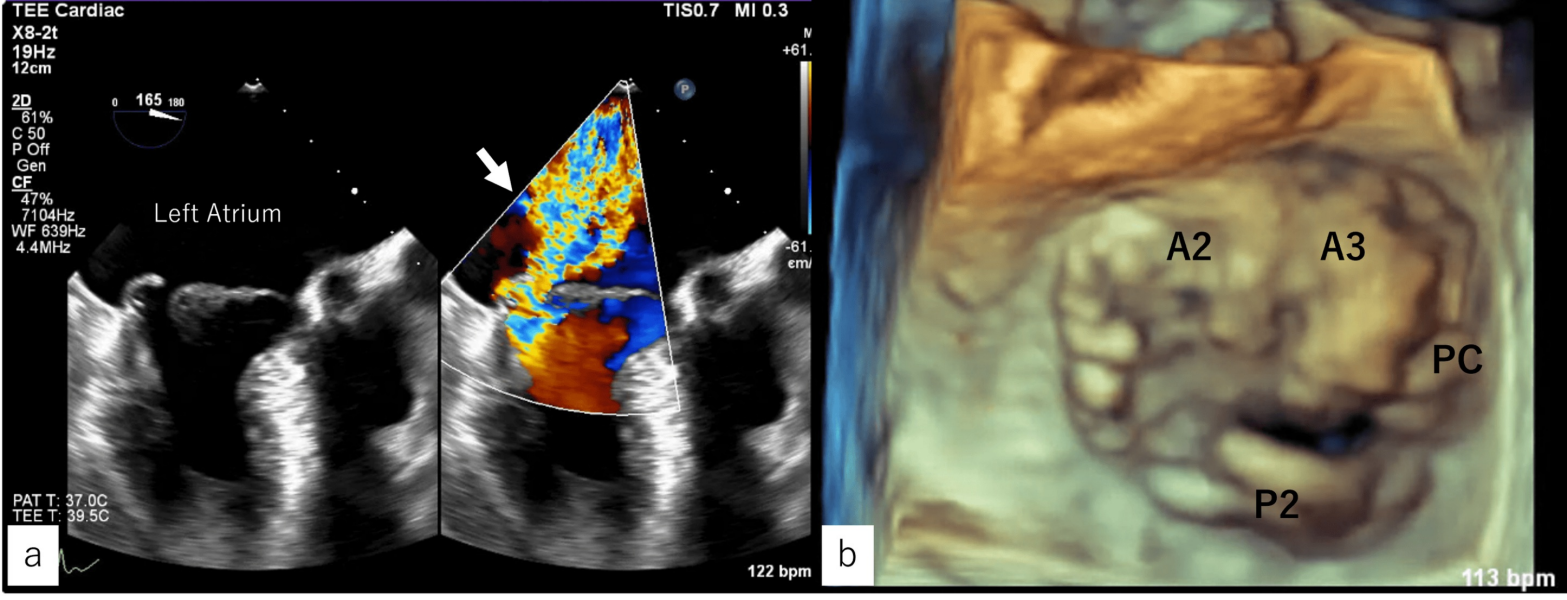
Preoperative transthoracic echocardiography (a) Transthoracic echocardiography demonstrating severe mitral regurgitation (arrow) with dilated left atrium. (b) Three-dimensional echocardiography showing prolapse of P2, A3, and the posterior commissure (PC).

Transthoracic echocardiography revealed an eccentric mitral regurgitant jet caused by P2, A3, and posterior commissure (PC) prolapse, with regurgitant volume of 87 mL and regurgitant fraction of 66%, indicating severe mitral regurgitation. The left atrium was also enlarged to 50 mm in diameter, and reversal flow to the pulmonary vein was also observed in the transesophageal echocardiography (Figure 2). 

The patient was recommended for surgical treatment due to symptomatic severe MR with a new onset of AF. For the severity of DM1, spirometry indicated a worsening restrictive ventilatory defect. Nine months prior to the surgery, forced vital capacity (FVC) was 2,770 mL (%VC: 71.2%), and forced expiratory volume in one second (FEV1)/FVC ratio was 79.1%, which decreased to FVC of 2,210 mL (%VC: 59.2%) and an FEV1/FVC ratio of 78.7% at the time of surgery. At the proposed time of hospital admission, he was able to walk 200 m with a walker. His grip strength was 5 kg on both hands. Short physical performance battery showed a total score of 10 points, including 1 point for side-by-side stand, 1 point for semi-tandem stand, 0 points for tandem stand, 2 points for gait speed test, and 4 points for chair stand test. He had no past medical history of general anesthesia.

Surgical procedure

Anesthesia was induced with midazolam (3 mg), fentanyl (200 µg), and rocuronium (30 mg), followed by intubation with an 8.0 mm endotracheal tube. Neuromuscular blockade was monitored using the train-of-four (TOF) technique. General anesthesia was maintained with propofol (150 mg/h) and remifentanil (0.05-0.2 µg/kg/min). The patient was placed in a left semi-lateral decubitus position. A 6 cm right mini-thoracotomy was performed at the fourth intercostal space. Cardiopulmonary bypass (CPB) was established via the right femoral artery and a single right femoral vein cannula. Mild hypothermia (30.0°C) was maintained during CPB. After cross-clamping the ascending aorta, antegrade crystalloid cardioplegia was administered to achieve cardioplegic arrest.

**Figure 3:**
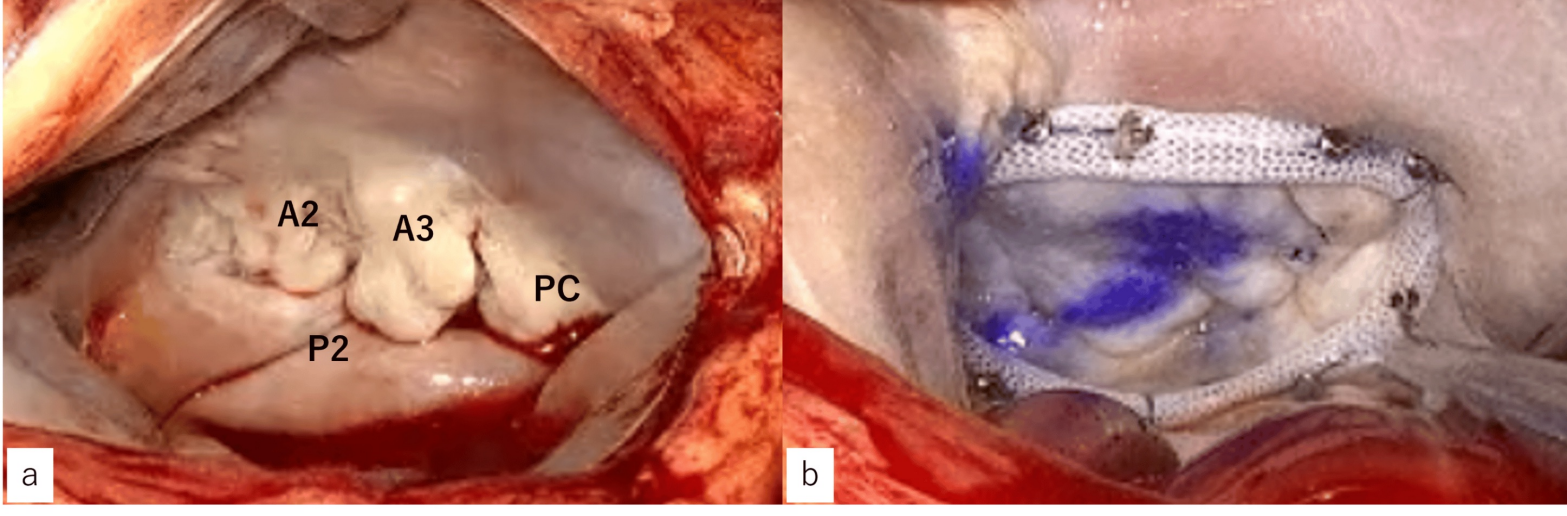
Intraoperative mitral valve findings before and after repair (a) Intraoperative view showing prolapse of P2 and A3 with involvement of the posterior commissure (PC). (b) Intraoperative view after mitral valve repair; the saline water test confirmed no residual leakage.

St. Thomas' Hospital II solution (Miotector; FUSO Pharmaceutical Industries, Ltd., Osaka, Japan) was used as the cardioplegic agent, with an initial dose of 30 mL/kg, followed by supplemental doses of 600 mL every 30 minutes. Under three-dimensional (3D) endoscopic guidance, the mitral valve was exposed via a right-sided left atriotomy. Cryoablation was performed using cryoICE™ (AtriCure, Inc., Mason, OH), isolating both pulmonary veins from the posterior left atrial wall to the mitral annulus. Inspection of the valve revealed extensive prolapse of P2, PC, and A3 (Figure 3A).

Mitral valve repair was performed using the "loop-in-loop" technique. First, a small loop was made with Gore-Tex CV-2 (W. L. Gore & Associates, Inc., Newark, DE), which was sutured to the posterior papillary muscle. Subsequently, CV-4 sutures were passed through the small loop and fixed to P2 and A3 to complete the artificial chordae plasty. Additionally, an edge-to-edge repair was performed for the prolapsed PC. The mitral annulus was then reinforced using a 34-mm Physio II ring (Edwards Lifesciences, Irvine, CA) secured with 2-0 Tefdexer sutures.

Saline water test confirmed the complete disappearance of regurgitation (Figure 3B). The left atriotomy was closed, and the left atrial appendage was excluded using a 45-mm AtriClip Pro2 (AtriCure, Inc., Mason, OH). After releasing the aortic cross-clamp, the patient was weaned from CPB under ventricular pacing. Intraoperative transesophageal echocardiography showed no residual MR. The chest was closed in the standard fashion (total operative time: 242 min; CPB time: 155 min; aortic cross-clamp time: 117 min). An additional 100 µg of fentanyl and 5 mg of rocuronium were required during the procedure (Video 1).

**Video 1:** Intraoperative video A3, P2, PC prolapse was repaired by artificial chordaes, edge-to-edge repair, and mitral annular plasty.

Postoperative course

Neuromuscular blockade was reversed with 200 mg (2.8 mg/kg) of sugammadex at the end of the surgery. For postoperative analgesia, a paravertebral block was performed, followed by continuous administration of 0.25% levobupivacaine via a patient-controlled analgesia pump at 7 mL/h. The patient regained consciousness approximately one hour after admission to the intensive care unit (ICU). However, due to discomfort and pain from the endotracheal tube resulting in patient-ventilator dys-synchrony, fentanyl was restarted at 50 µg/h (0.70 μg/kg/h) and subsequently maintained at 12.5 µg/h (0.18 μg/kg/h). As the patient's consciousness and respiratory status gradually improved, he was extubated 25 hours after returning to the ICU. High-flow nasal cannula therapy was required from postoperative day (POD) 2 to POD 5 for hypoxemia, which recovered with diuretic therapy using 20 mg of furosemide every six hours (Table 1).

**Table 1:** Perioperative respiratory function and its associated treatment Perioperative analgesic agents, blood gas and its associated treatment, is shown in the table. Administration of nasal high flow improved PaO_2_. PaO = partial pressure of arterial oxygen; PaCO_2_ = partial pressure of arterial carbon dioxide

Variables		POD0	POD1					POD2				POD3					POD4				POD5	
	Pre-op	Post-op	0:00	6:00	12:00	16:00	18:00	0:00	6:00	12:00	18:00	0:00	2:00	6:00	12:00	18:00	0:00	6:00	12:00	18:00	0:00	6:00
Body Weight (kg)	75.1 kg	78.7 kg	81.1 kg					80.5 kg				79.6 kg					76.9 kg				75 kg	
Urine Output (mL)		677 mL	1180 mL					1640 mL				3060 mL					3569 mL				1514 mL	
Respiratory Support		Ventilator				Mask							Nasal High Flow									Mask
Respirtory Support Details		PEEP 6 PS 12 FiO_2_ 0.5			PEEP 6 PS 6 FiO_2_ 0.4	Extubation	Mask 4 L					Mask 7 L	50L FiO_2_ 60%	50L FiO_2_ 50%		50L FiO_2_ 40%			40L FiO_2_ 40%		40L FiO_2 _35%	Mask 5 L
PaO_2_ (mmHg)		83.6	84.4	86.4	70.5		101.0	80.1	82.9	74.4	73.6	64.9		86.9	75.5	78.8	71.1	73.9	79.7	86.4	106.5	86.7
PaCO_2_ (mmHg)		47.3	39.1	30.4	25.7		32.5	40.5	40.5	31.6	36.5	36.3		36.6	38.5	36.7	43.0	39.0	36.0	37.6	36.6	35.9
SpO_2_ (%)		94	96	97	95		97	94	96	95	95	93		96	95	96	94	95	96	97	97	96
Dexmedetomidine (μg/h)		16 μg/h																				
Fentanyl (μg/h)		50 μg/h		9:00-16:0 12.5 μg/h																		
0.25% Levobupivacaine (mL/h)		7 mL/h																				
Furosemide (mg)												20 mg q6hrs					20 mg q6hrs				20 mg	
Sugammadex (mg)		200 mg																				

**Figure 4:**
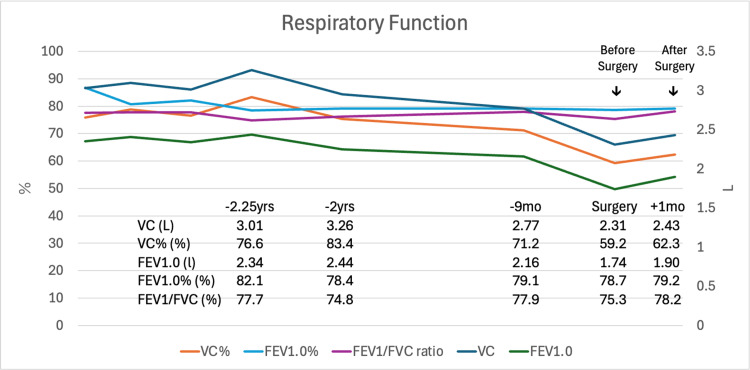
Perioperative spirometry Preoperative and postoperative spirometry showing no decline in respiratory function during follow-up. FEV1: forced expiratory volume in 1 second, FVC: forced vital capacity, FEV1/FVC ratio: also called the Tiffeneau-Pinelli index, which is a primary measurement in pulmonary function testing used to diagnose and differentiate between obstructive and restrictive lung diseases. It represents the percentage of a person's total air capacity that they can forcefully exhale in the first second of a breath.

The patient was transferred to the general ward on POD 8. Postoperative echocardiography confirmed no residual mitral regurgitant jet, with a mean pressure gradient of 1 mmHg and a mitral valve area of 3.53 cm². Although the patient was able to walk 100 m with a walker, he was transferred to a rehabilitation ward on POD 27 for further rehabilitation due to his desire to live by himself. Postoperative respiratory function testing performed one month after the surgery revealed an FEV1/FVC ratio of 79.1% and a %VC of 62.3%, indicating no decline in respiratory function compared with preoperative levels (Figure 4). 

Further, the short physical performance battery also recovered to baseline at one month after the surgery. Following intensive rehabilitation for muscle weakness and coordination of home care services, he was successfully discharged home, ambulatory, on POD 106. At the time of hospital discharge, his functional balance test was 49 points out of 57, which indicated good balance and a low risk of falling [4].

## Discussion

There are many emerging therapeutic approaches for myotonic dystrophies, including known small molecules, such as tideglusib, mexiletine, piltolisant, metformin, erythromycin, cannabinoids, theobromine, and caffeine. Nucleic acid-based therapies are another option, which include fatty-acid conjugation, monoclonal antibody conjugation, and peptide backbone or conjugation. Finally, genome/transcriptome engineering using Clustered Regularly Interspaced Short Palindromic Repeats and CRISPR-associated protein 9 (CRISPR-Cas9) technology is being tested in a preclinical study, which could remove dystrophia myotonica protein kinase (DMPK) CTG expansions at the DNA level [5]. CRISPR-Cas9 is a revolutionary gene-editing technology that allows scientists to precisely alter, add, or remove genetic material at specific locations within an organism's DNA. This system is recognized as a faster, cheaper, and far more accurate alternative to previous genetic engineering methods. Despite these improvements in therapeutic approach, management of patients with myotonic dystrophy under general anesthesia still requires careful attention.

In patients with myotonic dystrophy, use of depolarizing muscle relaxants is strictly contraindicated, as it can trigger sustained muscle spasm leading to airway obstruction [6]. As reported previously, a non-depolarizing muscle relaxant was safely used in the present case under TOF monitoring. It is also known that sensitivity to opioids is often heightened in patients with myotonic dystrophy, and postoperative opioid use is considered a significant factor that increases the complication rate [7]. Anesthetic management of patients with DM requires rigorous attention to complications such as myotonia and respiratory failure [8].

The present case involved severe mitral regurgitation complicated by new-onset atrial fibrillation and progressive restrictive respiratory dysfunction. Current consensus confirms that DM1 is characterized by slowly progressive restrictive respiratory failure, and FVC (% Predicted) is the factor most correlated with the disease severity [9]. Given that median sternotomy is associated with a significant decline in postoperative respiratory function [10], we selected the MICS approach to mitigate the risk of perioperative respiratory complications.

Compared with median sternotomy, MICS, which preserves the sternum, is reported to reduce postoperative ventilator duration and the incidence of complications, such as pneumonia and respiratory failure [11]. Preservation of the sternum facilitates thoracic volume changes by increasing the anterior and posterior diameter of the thoracic cavity during inspiration. The preservation of the xiphoid process is also essential in maintaining respiratory function, as it provides attachment of the diaphragm, which is a primary muscle for respiration. Further, accessory muscles, which are attached to the sternum, also assist with deep or forced breathing [12]. Although the patient had a worsening restrictive ventilatory defect preoperatively, the favorable respiratory outcome observed in this patient suggests that MICS may contribute to the preservation of respiratory function in selected DM1 patients.

Paravertebral block also reduced the requirement for opioids for wound analgesia [13]. Although fentanyl was restarted at 50 μg/h (0.70 µg/kg/h) to manage 8.0 mm endotracheal tube tolerance, the patient was successfully extubated 25 hours after the procedure. Following extubation, high-flow nasal cannula therapy at a flow of 50 L and FiO_2_ of 60% was required to manage impaired oxygenation associated with fluid refilling; however, the patient's recovery proceeded without major respiratory complications, such as reintubation or pneumonia. Although carbon dioxide (CO_2_) retention was not observed in the present case, the use of a high-flow nasal canula has also been reported to be useful in the elimination of CO_2_ in patients with myotonic dystrophy [14].

## Conclusions

Myotonic dystrophy is an autosomal dominant genetic disorder caused by the unstable expansion of nucleotide duplications. It is the most common and severe form of myotonic syndrome with an estimated incidence of 9.27 cases per 100,000. Clinical manifestations include muscle weakness, myotonia, and respiratory failure.

Although there are many emerging therapeutic approaches for myotonic dystrophies, including known small molecules, nucleic acid-based therapies, and genome/transcriptome engineering, management of patients with myotonic dystrophy under general anesthesia still requires careful attention to potential postoperative complications related to muscle weakness. Cardiac surgery, which commonly utilizes a median sternotomy, requires further attention to postoperative respiratory complications. Although the long-term outcome is yet to be determined, this case suggested that a minimally invasive approach, when combined with multidisciplinary perioperative management, can be safely performed in selected DM1 patients.
